# The Lipid Energy Model: Reimagining Lipoprotein Function in the Context of Carbohydrate-Restricted Diets

**DOI:** 10.3390/metabo12050460

**Published:** 2022-05-20

**Authors:** Nicholas G. Norwitz, Adrian Soto-Mota, Bob Kaplan, David S. Ludwig, Matthew Budoff, Anatol Kontush, David Feldman

**Affiliations:** 1 Harvard Medical School, Boston, MA 02115, USA; david.ludwig@childrens.harvard.edu; 2 Metabolic Diseases Research Unit, National Institute for Medical Sciences and Nutrition Salvador Zubiran, Tlalpan, CDMX 14080, Mexico; adrian.sotom@incmnsz.mx; 3 Citizen Science Foundation, Las Vegas, NV 89139, USA; bobotron@gmail.com; 4 New Balance Foundation Obesity Prevention Center, Boston Children’s Hospital, Boston, MA 02115, USA; 5 Lundquist Institute at Harbor-UCLA Medical Center, Torrance, CA 90502, USA; mbudoff@lundquist.org; 6 National Institute for Health and Medical Research (INSERM), UMRS 1166 ICAN, Faculty of Medicine Pitié-Salpêtrière, Sorbonne University, 75013 Paris, France; anatol.kontush@upmc.fr

**Keywords:** carbohydrate restriction, lean mass hyper-responder, LDL-cholesterol, lipoprotein lipase, HDL-cholesterol, triglyceride-rich lipoproteins, VLDL-cholesterol

## Abstract

When lean people adopt carbohydrate-restricted diets (CRDs), they may develop a lipid profile consisting of elevated LDL-cholesterol (LDL-C) and HDL-cholesterol (HDL-C) with low triglycerides (TGs). The magnitude of this lipid profile correlates with BMI such that those with lower BMI exhibit larger increases in both LDL-C and HDL-C. The inverse association between BMI and LDL-C and HDL-C change on CRD contributed to the discovery of a subset of individuals—termed Lean Mass Hyper-Responders (LMHR)—who, despite normal pre-diet LDL-C, as compared to non-LMHR (mean levels of 148 and 145 mg/dL, respectively), exhibited a pronounced hyperlipidemic response to a CRD, with mean LDL-C and HDL-C levels increasing to 320 and 99 mg/dL, respectively, in the context of mean TG of 47 mg/dL. In some LMHR, LDL-C levels may be in excess of 500 mg/dL, again, with relatively normal pre-diet LDL-C and absent of genetic findings indicative of familial hypercholesterolemia in those who have been tested. The Lipid Energy Model (LEM) attempts to explain this metabolic phenomenon by positing that, with carbohydrate restriction in lean persons, the increased dependence on fat as a metabolic substrate drives increased hepatic secretion and peripheral uptake of TG contained within very low-density lipoproteins (VLDL) by lipoprotein lipase, resulting in marked elevations of LDL-C and HDL-C, and low TG. Herein, we review the core features of the LEM. We review several existing lines of evidence supporting the model and suggest ways to test the model’s predictions.

## 1. Introduction

Carbohydrate-restricted diets (CRDs) hold promise for weight loss, and treatment of type 2 diabetes and other chronic health conditions [1]. However, this dietary strategy may cause elevated LDL-cholesterol (LDL-C), an important risk factor for atherosclerotic cardiovascular disease (ASCVD). Some studies report marked increases in LDL-C with consumption of a CRD [2,3,4,5,6,7]. However, others show no clinically meaningful increases [8,9,10,11,12,13,14,15]. Given the rise in popularity of CRD [16], as well as the persistent mortality burden of ASCVD [17], it is important to clarify the mechanistic understanding of the LDL-C elevation, and other alterations in lipid metabolism, in response to carbohydrate restriction. 

We recently reported data on a cohort of 548 individuals consuming a CRD, defined as <130 g/day (mean intake of 27 g/day). A hypothesis-naïve exploratory analysis identified body mass index (BMI) and the metabolic health markers, HDL-cholesterol (HDL-C) and triglycerides (TG), pre-CRD, as strong predictors of LDL-C increase. Specifically, BMI and TG-to-HDL-C ratio (TG/HDL-C) were each inversely associated with LDL-C change [18]. Otherwise stated, lean persons with low TG/HDL-C ratio (opposite to that characteristic of atherogenic dyslipidemia) were most likely to experience LDL-C increases with consumption of a CRD. We further characterized a subset of 100 “Lean Mass Hyper-Responders” (LMHR) who were leaner than non-LMHR participants and exhibited exceptional increases in LDL-C and HDL-C upon a CRD in the context of low TG (to mean levels of 320 mg/dL LDL-C, 99 mg/dL HDL-C, and 47 mg/dL TG on a CRD), despite no difference in pre-CRD LDL-C levels, as compared to non-LMHR. In those patients who have been tested, there were no notable genetic findings and the LMHR phenotype was reversible with reintroduction of carbohydrates [7,18].

The aim of this narrative review is to propose a mechanistic explanation for the LMHR phenotype, comprising the Lipid Energy Model (LEM), to inform prospective research. We first describe the core features of the model, with emphasis on the importance of lipoprotein lipase (LPL)-mediated lipolysis of triglyceride-rich lipoprotein (TGRL), including very low-density lipoprotein (VLDL). We consider several lines of evidence supporting the model and then propose future studies that could rigorously test the LEM. The research into this model may have important implications for the understanding of human lipid metabolism and cardiovascular disease. 

## 2. Core Concept of the Lipid Energy Model

### 2.1. Lipid Energy Model Overview

The central concept of the LEM is that, under conditions of carbohydrate-restriction and relatively low total adiposity, there is a greater reliance on fat as a metabolic substrate, as supplied by TGRL, including VLDL synthesized and secreted by the liver. Under these conditions, increased hepatic VLDL secretion is complemented by increased peripheral VLDL-TG lipolysis (which we will refer to as “VLDL turnover”), mediated by LPL, which results in the observed triad of high LDL-C, high HDL-C, and low TG. 

**Figure 1:**
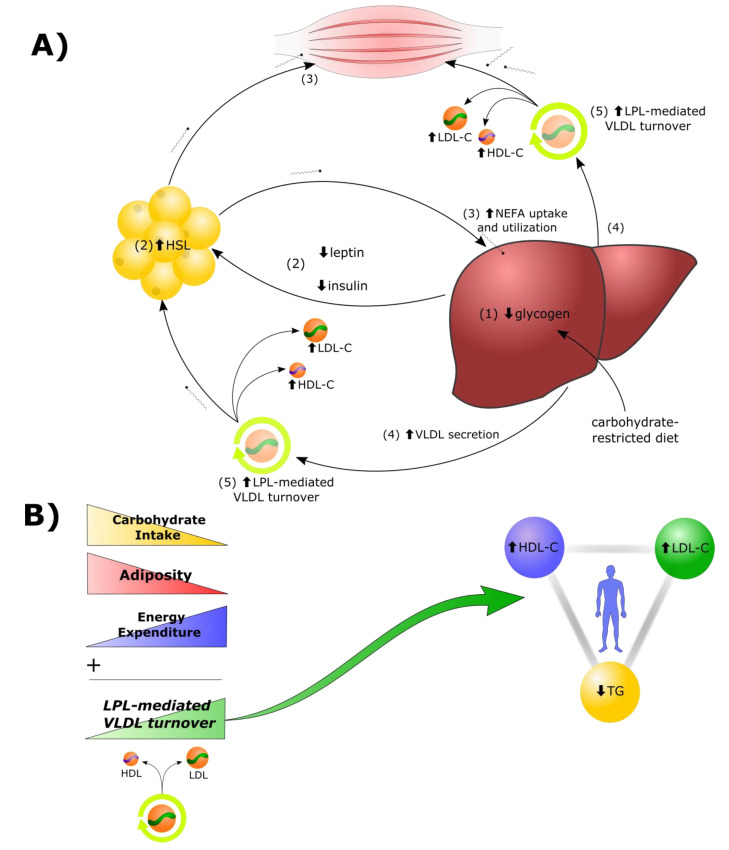
*The Lipid Energy Model*. (**A**) In the context of carbohydrate restriction, (1) glycogen depletion and (2) changes in circulating hormones stimulate hormone-sensitive lipase (HSL)-mediated secretion of non-esterified fatty acids (NEFA) by adipocytes to fuel oxidative tissues. (3) The liver captures circulating NEFAs and repackages them into triglycerides (TG), (4) secreted aboard VLDL. (5) Increased lipoprotein-lipase (LPL)-mediated VLDL turnover generates increased LDL-C and HDL-C. (Further details can be found in the main text.) The role of chylomicrons in the post-prandial state is presented later in the text. (**B**) The magnitude of carbohydrate restriction, adiposity, and energy expenditure each contribute, as independent variables, to the degree of LPL-mediated VLDL turnover and, thereby, to the magnitude of change of the triad components.

The proposed sequence of events is shown in Figure 1A:

1.  Reduction in dietary carbohydrates and depletion of hepatic glycogen stores results in a greater demand for fat as a metabolic fuel, to compensate for reduced glucose availability.
2.  Decreased insulin, leptin, and other changes to the hormonal milieu, result in increased hormone-sensitive lipase (HSL)-mediated lipolysis in adipocytes and greater secretion of non-esterified fatty acids (NEFAs) into the bloodstream.
3.  In addition to heightened use by tissues in the periphery, there is a greater rate of uptake of NEFAs by the liver. Under these conditions, there is a greater rate of synthesis of TGs from the increased fatty acid pool within hepatocytes.
4.  Increased rates of TG synthesis in the liver leads to increased rates of hepatic assembly and secretion of TG-rich VLDL.
5.  The increased VLDL secretion rates, in concert with greater LPL-mediated turnover of VLDL in peripheral tissues, and greater transfer of VLDL surface components (including free cholesterol) to HDL, result in higher plasma levels of LDL-C and HDL-C.

An important prediction of the model is that the magnitude of change in triad components is positively associated with reliance on energy supplied by VLDL-TG. Thus, as shown in Figure 1B, (i) greater restriction of carbohydrate, (ii) lower relative adiposity, and (iii) increased total energy expenditure (related to lean mass, physical activity level, and other factors) comprise independent variables that can be manipulated to test the model, as considered below. First, we will review the foundational literature upon which the LEM is based.

### 2.2. LPL Mediates the Exchange between TGRL Donors and HDL Acceptors

**Figure 2:**
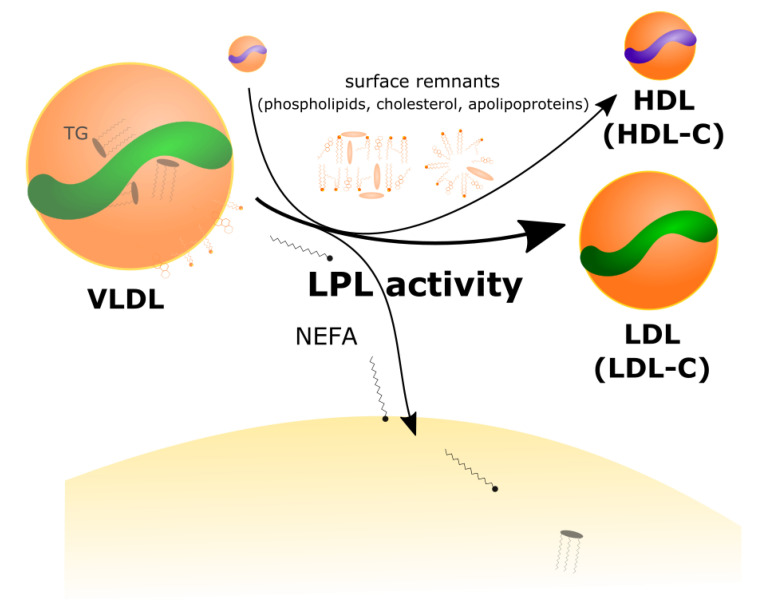
*Lipoprotein lipase-mediated increase in LDL-C and HDL-C.* In the presence of increased VLDL synthesis and secretion, lipoprotein lipase (LPL) activity liberates non-esterified fatty acids (NEFAs) for adipocytes and oxidative tissues. As TGs are lipolyzed, VLDLs shrink with loss of surface remnants (including cholesterol, phospholipids, and apolipoproteins) to HDL acceptor particles, and subsequent catabolism to LDL, resulting in increases in LDL particle mass, LDL-C, HDL particle mass and HDL-C.

Above, we introduced the idea that LPL-mediated VLDL turnover contributes to increases in LDL-C and HDL-C. This statement deserves some unpacking. The enzyme LPL is synthesized by peripheral tissues and anchored to endothelial cells in the capillary lumen. From there, LPL catalyzes the rate-limiting-step for the removal of TGs from TGRL, including chylomicrons in the post-prandial phase and VLDL in the fasted state. Focusing first on VLDL metabolism, LPL liberates NEFAs from TG contained within the core of VLDL to supply local tissues with substrate for storage (e.g., adipocytes) or oxidation (e.g., skeletal and cardiac muscle). Depletion of the core TG cargo of VLDL requires a complementary remodeling and shrinking of the surface area of the lipoprotein. VLDL surface components—including apolipoproteins, free cholesterol, and phospholipids—are thus transferred to HDL, resulting in increased HDL-C and LDL-C, as well as elevated total HDL and LDL particle mass (Figure 2). 

Studies in vitro have demonstrated that LPL activity and resultant VLDL catabolic rate are positively correlated with HDL-C [19]. Moreover, smaller lipid-poor HDLs act as preferential “acceptors” that develop into larger cholesterol-rich HDLs due to LPL-mediated VLDL-TG lipolysis [20,21]. This view is consistent with the observations that fat feeding in humans shifts HDL particle distribution towards larger cholesterol-rich HDLs [22,23] and that insulin resistance and plasma TGs are inversely associated with large cholesterol-rich HDLs [24]. 

Thus, LPL-mediated VLDL turnover is a process in which TG fuel is drained from VLDLs and, as the VLDL ‘shrinks’ into an LDL, the shed surface components are picked up preferentially by small lipid-poor HDLs. Stated concisely, the LEM emphasizes the donor–acceptor relationship between apoB-containing VLDL and apoA-I-containing HDL, a relationship that is relatively well-accepted in the literature [25]. However, this relationship is further expanded in the LMHR phenotype by the proposed increased rates of VLDL secretion (necessarily resulting in elevated LDL and LDL-C) and by the effect of low-carbohydrate, high-fat feeding on HDL dynamics, a topic to which we will now turn. 

## 3. The Role of HDL Particles in Lipid Energy Metabolism

### 3.1. High HDL Is Both a Cause and Consequence of Efficient TGRL Metabolism 

In the previous section, we introduced the idea that small lipid-poor HDLs are good acceptors for LPL-mediated release of surface components from TGRLs, including chylomicrons and VLDL. Therefore, one might expect that high-fat feeding would not only contribute to a shift in the HDL particle distribution to larger particles following LPL-mediated TGRL turnover [22,23], but also upregulate the production of new small HDLs to refresh the pool of acceptor particles and in anticipation of increased LPL-mediated release of TGRL surface remnants. 

In addition to direct hepatic apoA-I/HDL production, the intestine can produce small nascent HDL particles de novo [26,27,28]. Furthermore, chylomicrons, formed as the inevitable result of fat-feeding, provide a supply of apoA-I that can contribute to the formation of HDL particles following LPL-mediated VLDL turnover. Chylomicrons share a similar apolipoprotein profile with HDL, including apoA-I, a sizeable minority (up to 30–40%) of which is synthesized by the intestines prior to being trafficked in chylomicrons to systemic circulation [27,29]. Once in the bloodstream, LPL-mediated catabolism of chylomicrons transfers apoA-I preferentially to small lipid-poor protein-rich HDL. Given that apoA-I functions as an excellent biological detergent, this transfer of apoA-I can be thought of as conferring the propensity to “accept” TGRL surface components [25,30]. Small “acceptor” HDL particles can also be assembled de novo from apoA-I and other surface components released during LPL-mediated chylomicron lipolysis [31]. 

**Figure 3:**
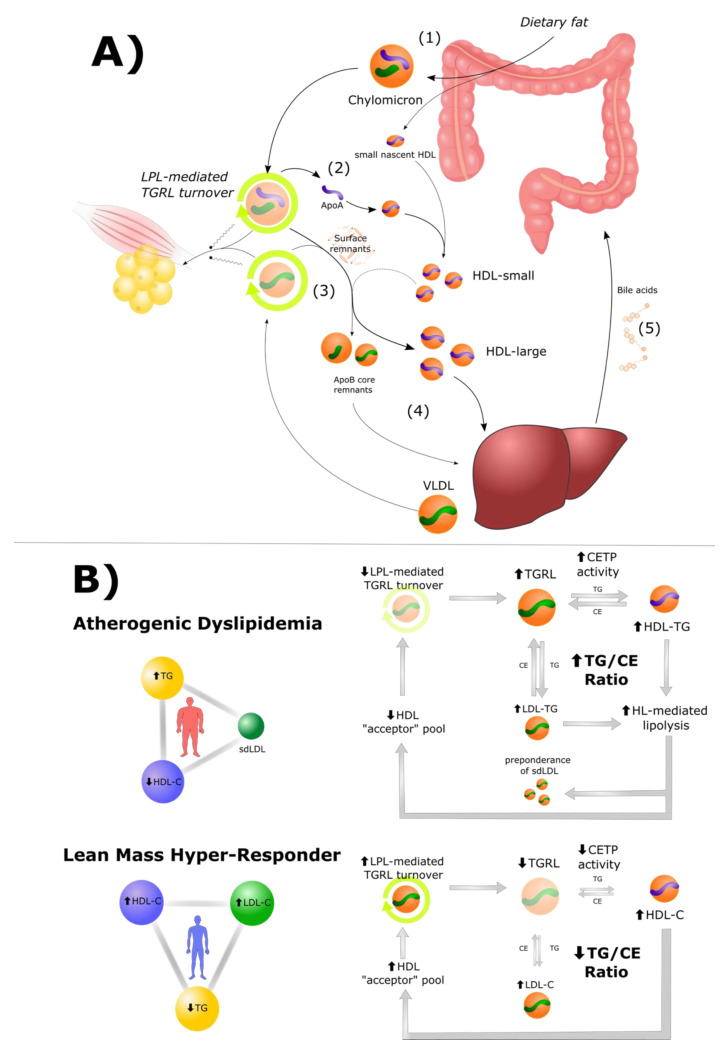
*High HDL is a result and cause of efficient lipoprotein lipase-mediated triglyceride-rich lipoprotein metabolism, and vice versa*. (**A**) (1) Ingestion of dietary fat contributes to production of small nascent HDL particles and apoA-I-containing chylomicrons. (2) Lipoprotein lipase (LPL)-mediated catabolism of chylomicrons yields lipid-free/lipid-poor apoA-I to further contribute to the pool of small HDL “acceptor” particles. (3) As LPL-mediated catabolism of triglyceride-rich lipoproteins (TGRLs, including chylomicrons and VLDLs) occurs, TGRLs release surface remnants. Surface remnants, bilayer structures including cholesterol, phospholipids, and apolipoproteins, are predominantly taken up by small acceptor HDL particles. (4) Through this process, lipoproteins, originating as TGRLs, remodel to intermediate-density lipoprotein (IDL) and finally to LDL and smaller chylomicrons and are returned to the liver. Large cholesterol-rich HDL (produced largely as a function of efficient LPL-mediated TGRL turnover) returns cholesterol to the liver, (5) to be used in various ways, such as the synthesis of bile acids. (**B**) The contrapositive is that low plasma HDL concentrations can be a result and cause of inefficient LPL-mediated TGRL turnover, a phenomenon that can be used to mechanistically contrast atherogenic dyslipidemia and the Lean Mass Hyper-Responder (LMHR) phenotype and involves cholesteryl ester transfer protein (CETP). In atherogenic dyslipidemia, insufficient turnover yields elevated TGRLs, driving CETP-mediated transfer of TGs to HDL and LDL particles (by heterotypic and homotypic exchange, respectively). This increases hepatic lipase (HL)-mediated lipolysis with ensuing depletion of HDL and production of small dense LDL (sdLDL). Decreases in the HDL acceptor pool perpetuates the cycle. This is in contrast to the elevated LPL-mediated TGRL turnover proposed in LMHR, in which low TGRL leads to a low TG/CE ratio in lipoproteins and low CETP activity, resulting in cholesterol-rich HDL (and low levels of HL-mediated lipolysis) and elevated HDL-C and LDL-C.

In summary, low-carbohydrate, high-fat feeding can promote intestinal secretion of apoA-I-containing particles (small nascent HDLs and chylomicrons), an adaptive response that can facilitate efficient LPL-mediated lipolysis of TGRL. In turn, elevated LPL-mediated TGRL turnover results in elevated circulating levels of larger HDLs and the subsequent efficient return of the cholesterol fraction [32] to the liver. In this way, HDL engages in a cycle that serves to couple digestion to energy demands: it accepts surface components upon TGRL lipolysis and returns cholesterol to the liver for bile acid synthesis to facilitate future fat digestion (Figure 3A). As is proposed by the co-author AK in the Reverse Remnant-Cholesterol Transport (RRT) theory, “high plasma HDL concentrations can therefore be a result and cause of efficient [LPL-mediated] clearance of TGRL” [25]. 

### 3.2. Cholesteryl Ester Transfer Protein (CETP) in Atherogenic Dyslipidemia and LMHR 

In the previous subsection, we explained how high HDL could be both a cause and consequence of efficient TGRL metabolism. To better understand how the converse could also be true as part of the LEM framework—that low HDL could be a cause and consequence of inefficient TGRL metabolism—it is instructive to consider the role of cholesteryl ester transfer proteins (CETPs). CETPs facilitate the exchange of core lipids, cholesteryl ester (CEs) for TGs, between lipoproteins [33]. Specifically, CETP is primarily bound to HDL [34] and facilitates the transfer of CE from apoA-I-containing HDL to TGRL and LDL, in exchange for TG from these apoB-containing particles. After receiving CE from HDL, these apoB-containing particles can traffic it to the liver or intestine in a pathway referred to as indirect reverse cholesterol transport [35,36].

However, given that CETP activity promotes the equimolar exchange of TG and CE, it is also appropriate to think about CETP from a TG-first framework, in line with the LEM. Indeed, CETP activity is largely driven by the availability of TGRL. Simply stated, higher levels of TG in apoB-containing particles drives increased CETP activity by increasing availability of TG substrate [37]. Conversely, in the context of low levels of TG in apoB-containing particles, there is little demand and little driving force for CETP activity. 

Conceptualizing of CETP in this way helps explain the profile of individuals with atherogenic dyslipidemia, typified by high serum TG, low HDL-C, and a preponderance of small dense LDL particles (sdLDLs). In atherogenic dyslipidemia, insufficient VLDL turnover relative to secretion (likely in part due to ApoCIII-mediated inhibition of LPL [38,39]) causes TGRL levels to rise and increases TGRL-TG, in favor of increased CETP activity. As a result, there are higher levels of CETP-mediated exchange [40,41,42,43,44], and HDL particles become relatively TG-rich and CE-poor (high TG/CE ratio), corresponding to reduced HDL-C levels. TG-rich HDLs are also prone to hepatic lipase-mediated lipolysis [45] (activity of hepatic lipase is also elevated in atherogenic dyslipidemia [46,47]) and ultimately accelerated HDL clearance by the liver and kidneys, further depleting HDL [45,48,49,50,51,52]. In brief, inefficient LPL-mediated VLDL turnover and increased VLDL levels, marked by elevated serum TG, drive higher rates of CETP-mediated transfer of TG to HDL, and consequently depletion of HDL-C and accelerated HDL catabolism.

The third characteristic of atherogenic dyslipidemia (in addition to elevated TG and diminished HDL-C) is a preponderance of sdLDLs, which can also be framed by our discussion of CETP. While HDL particles serve as the preferential CE donor (TG acceptor) for CETP-mediated exchange [53,54], TGRLs and their remnants that are elevated in atherogenic dyslipidemia also undergo CETP-mediated exchange in which they are the CE acceptor (TG donor) with other apoB particles, including LDL (this is “homotypic exchange,” as opposed to “heterotypic exchange” between apoA-I- and apoB-containing particles), resulting in TG-enriched LDL [35,55]. TG-enriched LDLs are, likewise, prone to hepatic lipase-mediated lipolysis, as with TG-rich HDL [56,57], resulting in sdLDL particles and the pattern B phenotype [58,59,60]. Moreover, transfer of CE to TGRL remnants, resulting from homotypic exchange generates particles that will eventually become LDL particles and in turn accept TG from, and donate CE to, another TGRL remnant, repeating the cycle. 

In summary, the extent of decreased LPL-mediated TGRL turnover rate and increased levels of TG substrate in apo-B-containing particles can drive enhanced CETP-mediated transfer of TG from TGRL to HDL and LDL, leading to depletion of HDL, as well as a preponderance of sdLDL, that defines atherogenic dyslipidemia. 

How would the scenario differ in LMHR? According to the LEM, the LMHR phenotype is characterized by a rapid rate of LPL-mediated TGRL turnover, exhibited by the presence of low plasma TG. Efficient turnover results in rapid shrinking of VLDL into CE-rich TG-poor LDL, and its shed surface components, including phospholipids and free cholesterol, that are acquired by HDL. HDL thus becomes enriched in cholesterol and CE, and HDL-C levels rise. Otherwise stated, efficient LPL-mediated TGRL turnover results in a diminution of TG and augmentation of CE in circulating Apo-A-I- and ApoB-containing lipoproteins (a low TG/CE ratio). This in turn results in low CETP activity and a greater proportion of larger CE-rich LDL and HDL and higher LDL-C and HDL-C. 

Putting the pieces of the puzzle together (Figure 3B), the LEM predicts that elevated CETP-mediated exchange, observed in people with atherogenic dyslipidemia, is not a cause, but a consequence, of inefficient LPL-mediated TGRL turnover. Elevated CETP-mediated exchange may then contribute to a vicious cycle of inefficient TGRL turnover, in part due to HDL deficiency. Borrowing from the Reverse Remnant-Cholesterol Transport (RRT) model in the contrapositive: low plasma HDL concentrations can therefore be a result and cause of inefficient [LPL-mediated] clearance of TGRL.

## 4. Adiposity and Lean Mass Influence Lipid Energy Dynamics

Having discussed some of the core features of the LEM, including the critical roles of LPL and HDL in VLDL-mediated turnover, we now turn to the question, “Why does the high LDL-C, high HDL-C, low TG lipid triad appear primarily in persons who are lean and/or athletic?” More specifically, what are the roles of adiposity and lean mass in lipid energy dynamics?

### 4.1. Obesity Impairs the Shift from Glucose to Fat Metabolism 

Given the relative paucity of data directly comparing people who are lean versus those with obesity on CRD, we will first draw from such comparative data in fasted subjects. Fasting provides a hormonal and metabolic state with parallels to a CRD, including reduced basal insulin level, depleted hepatic glycogen stores, and increased reliance on fat, relative to carbohydrate, for energy requirements. 

As early as 1977, Goschke observed that a six-day fast increased plasma NEFAs and ketones to a greater extent in subjects who are lean versus those with obesity. Plasma NEFAs and ketones in the lean participants were 22% and 30% higher, respectively, as compared to levels in those with obesity after six days of fasting [61], a particularly notable finding considering that plasma NEFAs tend to be higher among subjects with obesity at baseline or on a diet including carbohydrates. Similar results were produced more recently by Bak et al. in 2018, who showed that plasma NEFAs, glycerol (a gluconeogenic substrate), and ketones increased to a greater extent after a 72-h fast in subjects who were lean versus in those with obesity [62]. Furthermore, Wijngaarden et al. demonstrated that the shift from glucose to lipid oxidation is attenuated by obesity during a fast. In this study, over a 48-h fast, there was a 214% increase in lipid oxidation in lean subjects, as measured by indirect calorimetry, as compared to a 76% increase in those with obesity, with an accompanying decrease in resting energy expenditure observed in those with obesity only [63]. These studies each support the notion that metabolic flexibility and the shift towards a fat-based metabolism is impaired by obesity. 

### 4.2. Lean Body Mass and Adiposity Impact VLDL Secretion, Clearance, and Turnover

Whereas these fasting studies demonstrate leanness is associated with a greater upregulation of lipid oxidation, they do not examine VLDL dynamics directly. What determines VLDL secretion, clearance, and turnover rates? One telling human radiotracer study performed by Sondergaard et al. observed that lean body mass (LBM) was the strongest positive predictor of VLDL secretion [64]. As LBM is tightly correlated with metabolic rate, the authors interpreted these data as indicating that “VLDL-TG secretion is regulated by oxidative needs [derived from circulating TGRL].” Additionally, they observed that VLDL clearance rate was inversely related to total fat mass and insulin. Applied to the situation of lean, athletic persons with high energy demands on CRD, these tracer data predict elevated secretion rates of VLDL-TG to meet oxidative demand, along with rapid VLDL clearance and turnover rates, consistent with the LEM. 

Complementary evidence that adiposity impairs LPL-mediated VLDL turnover rates comes from the examination of reciprocal changes in VLDL and HDL surface lipids in response to cold exposure. Cold stimulates thermogenic brown and beige adipocytes to generate heat, energy from which can be provided by circulating VLDLs. In an elegant series of experiments in mice, Bartelt and colleagues [65] were able to demonstrate that LPL-mediated lipolysis of VLDL was critical to support heat production by thermogenic adipocytes and that this resulted in a reciprocal change of surface components across VLDL and HDL species. Importantly, when human subjects who were lean or obese were exposed to cold, changes in the HDL lipidome were only observed in the former. These data are consistent with the notion that, when persons with varying levels of adiposity are subjected to similar environmental circumstances that should induce LPL activity to meet energetic demands, the response is attenuated in those with obesity and accentuated in lean individuals.

### 4.3. Is There a “Threshold Effect” of Carbohydrate Intake on LDL-C Levels?

To summarize, the above human clinical data support the notions that excess adiposity impairs the shift to a fat-centric energy metabolism, in the context of carbohydrate restriction, and that increased oxidative demands can drive increases in VLDL secretion of TGRL. However, thus far, our explanation of the LEM would appear to imply a direct linear, or semi-linear, relationship between relative energy needs derived from fat, as compared to carbohydrate, and LDL-C levels. This is unlikely to be the case. Clinical anecdotes and case series suggest that the relationship between dietary carbohydrate intake as a percentage of energy needs and LDL-C levels may exhibit more of a “threshold” effect. For example, in a case series of five patients [18] on very low-carbohydrate diets (<25g/day), who tested negative for familial hypercholesterolemia, reintroduction of 50–100 g/day of carbohydrates decreased LDL-C levels between 30 and 72% of initial levels (mean decrease of 210 mg/dL from 428 mg/dL to 218 mg/dL), even though all individuals continued to consume a low carbohydrate diet of <130 g/day. 

There are several possible explanations for this carbohydrate threshold effect. First, there may be a point at which LDL uptake by hepatic LDL receptors at the liver becomes rate limiting. As LPL-mediated VLDL turnover rates and subsequent LDL formation increase above the point matched by removal by LDL receptors, LDL particle number and LDL-C levels would rise until production and removal kinetics reach a new steady state, asymptotically approaching LDL-receptor saturation. 

A second intriguing possibility is that depletion of hepatic glycogen stores could serve as a trigger for increased rates of VLDL secretion and peripheral LPL-mediated VLDL lipolysis. We propose that the co-occurrence of low insulin and low hepatic glycogen (Figure 1A) is necessary to induce increased rates of lipolysis in adipocytes, increased circulating NEFAs, and subsequent increased rate of hepatic uptake of NEFAs, secretion of VLDLs, and LPL-mediated VLDL turnover, thus increasing the pool of resulting LDL particles. 

The idea that depletion of hepatic glycogen may underlie a potential threshold effect is supported by rodent experiments in which a decline in the rate of glycogenolysis triggered hypoleptinemia, that was necessary, in addition to low insulin, to increase adipocyte lipolysis, circulating NEFAs, and NEFA uptake by hepatocytes [66]. In this seminal study, either prolonged fasting or blocking glycogenolysis was sufficient to induce hypoleptinemia. Furthermore, infusion of exogenous leptin to physiological levels was sufficient to reduce many of the observed physiological changes induced by fasting, including attenuating increases in plasma NEFAs, glycerol, ketones, and FGF21 (more on FGF21 in a subsequent section). Furthermore, investigation of the human fasting metabolome in persons without obesity confirm hypoleptinemia occurs in the early phases of fasting and that plasma leptin is inversely correlated with NEFA [67]. 

However, while some hormonal responses to fasting or carbohydrate restriction may be generalizable between species, humans may have adapted to longer periods of fasting and more prolonged dependency on fat as a primary fuel compared to rodents. Indeed, in hyperleptinemic rodents, increased NEFA uptake by hepatocytes is associated with hepatic steatosis and depletion of apoB-100 [66,68]. However, hepatic steatosis is not commonly reported in humans on CRD and has been shown to be beneficial for non-alcoholic fatty liver disease (NAFLD) [69]. It is therefore feasible that humans, who evolved to transition between carbohydrate and fat-based metabolic states, are evolutionarily equipped to export NEFAs taken up by hepatocytes in the form of VLDLs.

In conclusion, the possibility that combined low insulin and low leptin, secondary to hepatic glycogen depletion, are necessary to trigger increased NEFA secretion and uptake by hepatocytes is attractive in the context of the LEM because this would help explain: (i) the potential “threshold” effect (depletion of glycogen triggers hypoleptinemia) and also (ii) why the metabolic phenotype associated with the model presents preferentially in lean persons (leptin is elevated in obesity). Admittedly, the purported presence of a threshold-like relationship between carbohydrate restriction and LDL change remains to be confirmed; and, even if it exists, empirically demonstrating these mechanisms will be a major challenge for the LEM.

## 5. Lipoprotein Lipase Regulators 

We now turn to discussing key LPL regulators, including the apolipoprotein apoC-III, angiopoietin-like (ANGPTL) family of proteins, and FGF21. 

### 5.1. ApoC-III and Other Apolipoproteins

Among the exchangeable apolipoproteins, apo-CIII is a strong negative regulator of LPL. ApoC-III is elevated in metabolic syndrome [70], diabetes [71] and even young “metabolically unhealthy” lean individuals [72]. In addition, its concentration correlates positively with serum TG [73]. Regarding dietary modulation, carbohydrate intake is positively associated with apoC-III in several studies [74,75,76,77] and isocaloric carbohydrate restriction (<30g/day), in subjects with NAFLD, lowers apo-CIII [78]. Given these data, and its strong association with ASCVD [79], pharmacological strategies to lower apo-CIII are being explored for the reduction of residual cardiovascular risk [80]. The LEM predicts low levels of TGRL apo-CIII in lean persons on CRD, with implications for the possibility of ASCVD risk attenuation in LMHR. It is also worth acknowledging that other transferable apolipoproteins, including the LPL inhibitor apo-CI, and the LPL activators apo-CII and apo-AV, may also have a particular profile in LMHR. However, the complexities of whether changes in levels of these proteins are a cause or consequence of metabolic dysfunction, and how they interact with dietary macronutrient composition, are beyond the scope of this manuscript and should be the topic of future exploratory investigations.

### 5.2. ANGPTL Proteins 

The ANGPTL family of LPL inhibitory proteins, consisting of ANGPTL 3, 4, and 8, constitute an elegant coordinated system that directs NEFA to adipose tissue during postprandial periods and to skeletal and cardiac muscle during fasting periods. The system operates as follows: ANGPTL4 is expressed locally at adipocytes and is upregulated in the fasted state [81,82], thereby downregulating LPL activity at adipocytes and reserving circulating NEFA for skeletal and cardiac muscle. In the fed state, ANGPTL4 activity decreases and ANGPTL3 and 8 are secreted by the liver and work systemically to direct NEFA away from these lean tissues and toward adipocytes for storage. Since ANGPTL3 expression is largely independent of nutrition status [83], and requires activation by ANGPTL8, which is induced by feeding, the system can be simplified as follows: ANGPTL4 increases and ANGPTL8 decreases in the fasted state to drive TG fuel to oxidative tissue, whereas following mixed diet feeding ANGPTL4 drops and ANGPTL8 levels increase to promote fat storage. 

The ANGPTL3-4-8 model [84] has been influential in the development of the LEM, which predicts chronically low ANGPTL8 in lean persons consuming CRDs. This prediction is bolstered by the findings that BMI positively correlates with ANGPTL8 [85] and that ANGPTL8 is induced by insulin [86]. Reduced levels of ANGPTL8 in LMHR could be one mechanism by which skeletal and cardiac myocytes maximize their capacity to oxidize TG from circulating TGRL. Predicting levels of ANGPTL4 is more complicated as, while BMI is positively associated with ANGPTL4 [87], it is induced by NEFA-responsive peroxisome proliferator activated receptor (PPAR) transcriptional regulators [88]. Thus, focusing specifically on the ANGPTL4 system, there are two opposing possibilities: that chronically elevated ANPGTL4 helps direct NEFA to oxidative tissues or that decreased ANGPTL4 permits rapid replenishment of depleted adipocytes in LMHR and that NEFAs flux from VLDLs through adipocytes and HSL-mediated lipolysis into oxidative tissue at a high rate. Investigating ANGPTL dynamics, in addition to radiotracer studies, could help parse between these two possibilities. 

### 5.3. FGF21

Finally, the role of fibroblast growth factor 21 (FGF21) in VLDL secretion and LPL-mediated VLDL turnover warrants consideration. FGF21 is a hepatokine and myokine induced downstream of PPAR-α in response to states of increased lipid energy demand and circulating NEFAs: states including fasting in humans [89], exercise [90], and a ketogenic diet in mice [91]. FGF21, in turn, regulates the expression of a wide array of genes important in ketosis and lipid oxidation, including ANGPTL4 [91]. Interestingly, in mice, FGF21 knockdown via short hairpin RNA contributed to hypertriglyceridemia that was not exacerbated by pharmacological inhibition of LPL, suggesting FGF21 may positively regulate peripheral LPL activity [91]. 

One could hypothesize that carbohydrate restriction and fat-adaptation in lean subjects causes increased lipolysis and circulating NEFAs, which activate PPAR-α to induce FGF21 and upregulate ketogenesis, lipid oxidation, and LPL-mediated VLDL turnover. This “*NEFA-PPAR-FGF21-LPL*” axis is particularly intriguing in light of thinking that obesity and related diseases may represent an “FGF21-resistant” state analogous to insulin resistance [92,93,94,95] and with paradoxically high FGF21, and could operate alongside promising efforts to develop FGF21-directed therapy to treat atherogenic dyslipidemia, diabetes, and NAFLD [96,97]. Exploring FGF21 dynamics, as well as the FGF21/FGF1 ratio that has been proposed to regulate lipolysis [98,99] (and even the possible impact of FGF21 on carbohydrate and sugar taste preferences [100] and alcohol consumption [101]), in LMHR as part of the LEM, could provide insight into hormonal mechanisms of metabolic disease.

Admittedly, interpretation of the literature on FGF21 is complicated by the fact that the hormone may serve different signaling roles in rodents and in humans and that serum measures of FGF21 in humans may not reflect levels of downstream signaling given varying degrees of FGF21 sensitivity. For example, weight loss with carbohydrate reduction may result in lower FGF21, associated with improvements in hepatic insulin sensitivity [95]. In brief, investigating FGF21 levels and downstream signaling in LMHR could provide insight more broadly applicable to human metabolism. 

## 6. Testable Predictions of the Lipid Energy Model

The aim of this review is to present an explanation of the LMHR phenotype, with testable hypotheses to guide future investigation. We here provide our “wish-list” of future experiments that could help to evaluate and/or evolve the LEM.

(i) **Tracer study:** Kinetic studies using stable isotope-labeled tracer methods could be employed to track (1) hepatic NEFA uptake, (2) hepatic VLDL-TG/VLDL-P secretion (3) VLDL peripheral turnover, and (4) free cholesterol transfer from TGRL to HDL.
  - The LEM predicts increased rates of hepatic NEFA uptake, VLDL-TG/VLDL-P secretion and turnover, and transfer of free cholesterol from TGRL to HDL, in lean metabolically healthy persons on CRD, as compared to metabolically unhealthy subjects or those with obesity.

(ii) **“Gym hypothesis:”** If the LMHR phenotype is a generalizable metabolic phenomenon, it should be reproducible. This possibility could be assessed with a dietary crossover study including a group of lean, athletic persons, and overweight non-diabetic controls consuming mixed diets. Both groups would undergo a controlled four-week weight-maintenance ketogenic diet and a controlled mixed diet to see if the LMHR phenotype could be induced and reversed.
  - The LEM predicts implementation of a CRD in lean metabolically healthy persons with high aerobic energy demands would reproducibly induce a LMHR or LMHR-like lipid profile. The LEM also predicts increases in exercise load would increase LDL-C and HDL-C in present (or induced) LMHR when controlling for weight and carbohydrate intake.

(iii) **Carbohydrate titration crossover:** To establish the nature of the relationship between carbohydrate intake as percent of total energy intake and LDL-C levels, it would be ideal to perform a crossover study including weight-stable LMHR in which calories from fat are traded for those from carbohydrates in increments of ~5% caloric intake.
  - The LEM predicts a threshold or bi-phasic relationship between carbohydrate intake and LDL-C, in which VLDL secretion, and LDL-C levels (as well as lipolytic transfer of free cholesterol from TGRL to HDL) decrease in correspondence with elevated hepatic glycogen stores and plasma insulin and leptin.

(iv) **Genetic heterogeneity:** Even if the LMHR phenotype is reproducible, there will still likely be a meaningful degree of heterogeneity in LDL-C response to CRD among subjects with similar levels of adiposity. It would be informative to perform a cross-sectional analysis of lean persons consuming a CRD with comprehensive genetic testing of genes relevant to the model, including those coding for the LDL-receptor, ApoB, PSCK9, CETP, LPL and its regulatory proteins (GPIHBP1, ANGPTL3, 4, and 8, ApoCI-III, ApoAV, FGF21), and more.
  - The LEM predicts inverse correlations between adiposity and LDL-C and HDL-C changes on CRD that would be relatively generalizable, but that the strength of the associations would be modified by gene-environment interactions that could meaningfully advance the LEM and integrate the model with present understanding of ASCVD pathology.

(v) **LPL regulator analysis:** It would be informative to measure levels of specific LPL regulators to gain insight into the possible mechanisms behind NEFA and TGRL trafficking.
  - The LEM predicts low levels of apo-CIII, ANGPTL8, and 3–8 complex. ANGPTL4 levels may be depleted or elevated in LMHR, with implications on whether NEFA are directed from TGRL directly to oxidative tissue or first trafficked through adipocytes. FGF21 may be elevated, although this prediction is complicated by the possible differing roles of FGF21 in rodent models and humans, and by the suggestion of FGF21 resistance in humans with obesity. Nevertheless, interrogating the possibility role of FGF21 signaling in LMHR may prove an informative line of investigation and one that provides broader clarification on FGF21 in human metabolism.

(vi) **Evaluation of risk:** While not directly related to the LEM itself, an important forthcoming line of inquiry regards an assessment of the degree to which the LMHR profile is associated with increased progression of atherosclerosis. The conservative clinical perspective is to assume that the high levels of apoB-containing lipoproteins present in LMHR are atherogenic and require treatment with lifestyle modification and/or pharmacotherapy, without exception. However, to properly provide evidence-based recommendations for this unique subgroup of patients we must systematically study them. A prospective study assessing atherosclerotic progression in 100 LMHR by coronary computed tomographic angiography, recently launched from the Lindquist institute, will provide data which will be informative regarding the clinical care of LMHR. However, given the absence of comparator coronary computed tomographic angiography data, and the fact that clinically relevant ASCVD can take years to decades to develop, there is much work to be done to assess the absolute clinical risk of the LMHR profile.

Concisely stated, the LEM provides a mechanistic hypothesis explaining the inverse association between BMI and LDL-C and HDL-C change that can occur in individuals adopting CRD in the context of low TG, as well as the associated LMHR phenotype. Critical future lines of investigation include:

- *Those that could evaluate key elements of the LEM*: Demonstrating increased hepatic VLDL secretion and LPL-mediated peripheral VLDL turnover in LMHR (tracer study) and demonstrating that the LMHR phenotype is relatively reproducible (gym hypothesis).
- *Those that would help to refine the LEM*: By better characterizing the dose-response relationship between carbohydrate intake and LDL-C change (carbohydrate titration crossover), describing the putative contribution of genotype (genetic heterogeneity), and defining the endocrinological mechanisms by which LPL activity is regulated in lean persons on CRD (LPL regulator analysis).
- *Those that would evaluate the atherosclerotic risk* associated with the LMHR phenotype (evaluation of risk, ongoing).

## 7. Conclusions

The Lipid Energy Model could explain the observed inverse association between BMI and LDL-C change in the context of carbohydrate restriction, as well as the Lean Mass Hyper-Responder phenotype. It provides specific and testable predictions that should be assessed in future studies. Insights gained from these studies will help us better understand the mechanisms behind LDL-C change on low-carbohydrate diets and may advance our knowledge of lipid and lipoprotein dynamics across human metabolic states. 

## Data Availability

Not applicable.

## Abbreviations

ASCVD	atherosclerotic cardiovascular disease
ANGPTL	angiopoietin-like proteins
BMI	body mass index
CE	cholesteryl ester
CETP	cholesteryl ester transfer protein
CRD	carbohydrate-restricted diet
FGF21	fibroblast growth factor 21
HDL-C	HDL-cholesterol
HSL	hormone-sensitive lipase
IDL	intermediate-density lipoprotein
LBM	lean body mass
LDL-C	LDL-cholesterol
LEM	Lipid Energy Model
LMHR	lean mass hyper-responders
LPL	lipoprotein lipase
NAFLD	non-alcoholic fatty liver disease
NEFA	non-esterified fatty acids
PPAR	peroxisome proliferator activated receptor
RRT	Reverse Remnant-Cholesterol Transport
sdLDL	small dense LDL
TG	triglycerides
TGRL	triglyceride-rich lipoprotein
VLDL	very low-density lipoprotein
